# Elevated Serum Triglyceride Levels in Acute Pancreatitis: A Parameter to be Measured and Considered Early

**DOI:** 10.1007/s00268-022-06533-w

**Published:** 2022-03-30

**Authors:** Nils Jimmy Hidalgo, Elizabeth Pando, Piero Alberti, Laura Vidal, Rodrigo Mata, Nair Fernandez, Maria Jose Gomez-Jurado, Cristina Dopazo, Laia Blanco, Stephanie Tasayco, Xavier Molero, Joaquim Balsells, Ramon Charco

**Affiliations:** 1 Universitat Autonoma de Barcelona, Department of Hepato-Pancreato-Biliary and Transplant Surgery, Hospital Universitari Vall d’Hebron, Passeig de la Vall d’Hebron, 119-129, 08035 Barcelona, Spain; 2 Department of Gastroenterology, Hospital Universitari Vall d’Hebron, Barcelona, Spain

## Abstract

**Background:**

The value of serum triglycerides (TGs) related to complications and the severity of acute pancreatitis (AP) has not been clearly defined. Our study aimed to analyze the association of elevated levels of TG with complications and the severity of AP.

**Methods:**

The demographic and clinical data of patients with AP were prospectively analyzed. TG levels were measured in the first 24 h of admission. Patients were divided into two groups: one with TG values of<200 mg/dL and another with TG≥200 mg/dL. Data on the outcomes of AP were collected.

**Results:**

From January 2016 to December 2019, 247 cases were included: 200 with TG<200 mg/dL and 47 with TG≥200 mg/dL. Triglyceride levels≥200 mg/dL were associated with respiratory failure (21.3 vs. 10%, *p*=0.033), renal failure (23.4 vs. 12%, *p*=0.044), cardiovascular failure (19.1 vs. 7.5%, *p*=0.025), organ failure (34 vs. 18.5%, *p*=0.02), persistent organ failure (27.7 vs. 9.5%, *p*=0.001), multiple organ failure (19.1 vs. 8%, *p*=0.031), moderately severe and severe AP (68.1 vs. 40.5%, *p*=0.001), pancreatic necrosis (63.8 vs. 34%, *p*<0.001), and admission to the intensive care unit (27.7 vs. 9.5%, *p*=0.003). In the multivariable analysis, a TG level of≥200 mg/dL was independently associated with respiratory, renal, and cardiovascular failure, organ failure, persistent organ failure, multiple organ failure, pancreatic necrosis, severe pancreatitis, and admission to the intensive care unit (*p*<0.05).

**Conclusions:**

In our cohort, TG≥200 mg/dL was related to local and systemic complications. Early determinations of TG levels in AP could help identify patients at risk of complications.

**Supplementary Information:**

The online version contains supplementary material available at 10.1007/s00268-022-06533-w.

## Introduction

Acute pancreatitis (AP) is a complex inflammatory disease that affects the pancreas, peripancreatic tissue, and distant organs [1]. The worldwide incidence is approximately 70 cases per 100,000 individuals [2], with a growing number of hospitalizations every year in the world [3, 4].

The clinical course of AP can range from relatively mild and self-limited disease with uneventful and brief hospitalization [5] to severe disease (15–20%), complicated by persistent or multisystem organ failure [6–8]. According to the revised Atlanta classification, severity is determined by local or systemic complications and can be classified into three groups: mild, moderately severe, and severe [8]. Mortality continues to be high, with values between 10 and 39%, in severe forms of the disease [9–11].

The main causes of AP are gallstone disease (40–60%) [12] and alcohol abuse, among other factors [13]. It has also been observed that elevated levels of serum triglyceride (TG) are commonly present in the early stage of acute biliary or alcoholic pancreatitis, but its clinical significance is still unknown. Only a few studies have observed the relationship between elevated levels of TG and complications in AP, with contradictory results [14–17].

Research on new risk factors for complications and mortality in AP is still a topic of interest [18, 19]. At this point, the values of TG in relation to local and systemic complications and the severity of AP have not been clearly defined. Our study aimed to analyze the association of elevated TG levels with complications and severity of AP.

## Materials and methods

### Study design

A prospective single-cohort observational study of adult patients diagnosed with AP in a third-level referral center was designed to evaluate the role of elevated levels of TG in AP patients.

### Inclusion criteria

Patients≥18 years of age with AP were included according to the definitions of Atlanta 2012 [8]. The diagnosis of AP requires two of the following three features: (1) Typical abdominal pain, (2) Serum amylase or lipase more than 3 times the normal values, and (3) Contrast computed tomography (CT) with radiological findings suggestive of pancreatitis. When CT was not performed, pancreatic magnetic resonance imaging (MRI) and abdominal ultrasound (US) with signs of pancreatitis were admitted. Patients underwent the measurement of TG levels in the first 24 h of admission.

### Exclusion criteria

Patients with AP whose etiology was hypertriglyceridemia (HTG) syndrome, patients with another major disease coexisting with AP (upper gastrointestinal bleeding that required an invasive endoscopic, radiological, or surgical procedure or that involved duodenal perforation or perforation of the bile duct), patients with another pathology not related to AP (periampullary neoplasia or neoplasia of the proximal or middle third of the biliary tract, or serious infectious pathology), transferred patients, and patients who arrived to the emergency department more than 72 h after the onset of symptoms.

These inclusion and exclusion criteria reduce the bias of including patients with prolonged disease, avoid alterations in TG values that can occur after 48 h due to other factors (such as fasting or the administration of parenteral nutrition), and analyze the role of TG levels as an early predictor of complications. Applying these criteria, we performed a subanalysis to determine whether the time from the onset of symptoms to admission could be a confounding factor, and we found no significant association with outcomes of acute pancreatitis.

### Management of AP

According to the international guidelines [20, 21], our institutional management protocol of AP initially included fluid therapy according to the patient characteristics with a goal of a urinary output of more than 0.5 ml/kg/hour, based on ringer lactate and isotonic sodium chloride solutions. No empirical antibiotics were used. When severe AP was suspected, the patient was referred to the ICU team for management and counseling.

### Triglyceride determination and group division according to TG levels

Serum TG levels were measured in the first 24 h after admission by enzymatic techniques with spectrophotometric methods (Beckman Coulter Method). In our laboratory, the normal reference intervals are 43–200 mg/dL, regardless of the sex and age of the patient.

We divided the sample based on the classification of the National Cholesterol Education Program-Adult Treatment Panel III (NCEP-ATP III) [22], in which patients with TG levels≥200 mg/dL are considered to have high TGs. We also generated ROC curves of TG values and AP outcomes to identify the best cutoff point (Youden's index), and we obtained values similar to the reference value. We divided the patients into two groups for our study: one with TG values<200 mg/dL and another with TG≥200 mg/dL (Supplementary Material Figures 1–5).

### Variables analyzed

Demographic data, including age, sex, and body mass index (BMI), were collected from all patients in the study. Previous diseases such as diabetes mellitus, high blood pressure, cardiovascular disease (myocardial infarction, heart failure, cardiac arrhythmia), chronic lung disease (obstructive or hypertensive pulmonary disease), and chronic kidney disease were recorded. The risk of patients according to the American Society of Anesthesiologists (ASA) classification was also recorded.

We collected vital signs upon admission, complete blood counts, complete metabolic profiles, liver function test results, serum amylase levels, and serum TG levels.

We also collected data on admission to the intensive care unit (ICU), the length of total hospital stay, and mortality during admission or up to 90 days after discharge.

#### Pancreatic or peripancreatic complications

Local complications evaluated were pancreatic and extrapancreatic necrosis, infection or necrosis, and the need for invasive procedures to treat necrosis.

*Pancreatic necrosis* was defined as the absence of enhancement in pancreatic tissue after contrast-enhanced CT or the presence of extrapancreatic fat necrosis.

*Infectious pancreatic necrosis (IPN)* was defined as a positive culture for microorganisms after necrosectomy or interventional (radiological or endoscopic) drainage. We also collected data on the need for an interventional radiological procedure, an endoscopic procedure, or surgical intervention.

#### Systemic complications

The systemic complications analyzed were respiratory failure, cardiovascular failure, acute renal failure, organ failure, persistent organ failure, multiorgan failure and systemic inflammatory response syndrome (SIRS).

*Organ failure* is the failure of an essential system in the body defined using the Marshall scoring system [23] as a score of 2 or more points for one of three organs (renal, cardiovascular, or respiratory). *Multiple organ failure* involves two or more organ systems, and p*ersistent organ failure* is defined as organ failure prolonged for 48 h or more.

*SIRS* is defined by the satisfaction of any two of the following criteria: temperature>38 or<36 °C, heart rate>90 beats/minute, respiratory rate>20 breaths/minute or partial pressure of CO2<32 mmHg, leucocyte count>12,000 or<4000/microliters or>10% immature forms or bands [24].

*Mortality* was defined as a fatality event occurring during admission or up to 90 days after discharge.

#### Severity classification and scores

The classification of the severity of AP of the patients was based on the 2012 revision of the Atlanta Classification [8], which is as follows: mild disease, moderately severe disease, and severe disease, according to the presence of organ failure and multiorgan failure and local complications.

The radiological severity of AP was assessed using the classical CT severity index classification [25] and the modified CT severity index [26, 27]. The Acute Physiology and Chronic Health Evaluation II (APACHE II) score [28] was also calculated for each patient at admission.

### Statistical analysis

We performed the Chi-square test or Fisher’s exact test to analyze qualitative variables. For normal distributions, the quantitative variables were compared by Student’s t test for two groups and skewed data, and the nonparametric test used was the Mann–Whitney *U* test. We performed multivariable logistic regression to evaluate the association between TG levels≥200 mg/dL and other risk factors for each outcome of AP. A value of *p*<0.05 was considered statistically significant. Statistical analysis was performed using the commercial software SPSS version 20 (SPSS Inc., Chicago, Illinois).

### Ethics

Following the Helsinki Declaration principles for human investigations, our local Ethical Committee approved the present study and the prospective database (PR-AG 328/2017). All participants signed informed consent to participate in our prospective register.

## Results

From January 2016 to December 2019, a total of 350 patients with AP were diagnosed. We excluded nine transferred patients, 54 patients arriving to the emergency department with more than 72 h from the onset of abdominal pain, three patients with HTG as a syndrome, and 37 patients in whom the determination of the TG level was not possible. Finally, 247 patients were included in the study.

Two hundred out of 247 (81%) patients had TGs<200 mg/dL, and 47 (19%) had TGs≥200 mg/dL. The mean TG levels were 111.24±36.88 mg/dL vs. 339.68±177.88 for the TG<200 mg/dL group and TG≥200 mg/dL group, respectively (*p*<0.001).

The baseline characteristics of the entire cohort are listed in Table 1. No significant differences were found between groups regarding previous comorbidities, and ASA classification, except for age and sex.

**Table 1:** Demographic characteristics of acute pancreatitis according to TG levels group

	All cohort	TG<200 mg/dL	TG≥200 mg/dL	*p*
	*N*=247	*N*=200	*N*=47	
Age, mean±SD	65.23±18.11	66.75±18.63	58.81±14.18	0.001
Male sex, *N* (%)	140 (56.7)	106 (53)	34 (72.3)	0.016
BMI Kg/m^2^, mean±SD	27.96±5.1	27.77±5.16	28.7±4.78	0.239
*Previous Diseases, N (%)*				
Diabetes	58 (23.5)	45 (22.5)	13 (27.7)	0.453
Higher blood pressure	141 (57.1)	115 (57.5)	26 (55.3)	0.786
Cardiovascular disease	61 (24.7)	52 (26)	9 (19.1)	0.327
Lung disease	38 (15.4)	29 (14.5)	9 (19.1)	0.427
Chronic kidney disease	23 (9.3)	21 (10.5)	2 (4.3)	0.266
Obesity	73 (30.5)	54 (27.7)	19 (43.2)	0.044
Dyslipidemia	84 (34)	63 (31.5)	21 (44.7)	0.086
Previous pancreatitis, *N* (%)	49 (25.7)	36 (23.2)	13 (36.1)	0.111
ASA (III, IV), *N* (%)	94 (38.1)	75 (37.5)	19 (40.4)	0.710
*Pancreatitis etiology, N (%)*				<0.001
Biliary	143 (57.9)	126 (63)	17 (36.4)	0.001
Alcoholism	45 (18.2)	26 (13)	19 (40.4)	<0.001
Idiopathics	39 (15.8)	32 (16)	7 (14.9)	0.852
Post-ERCP	8 (3.2)	6 (3)	2 (4.3)	0.650
Other	12 (4.9)	10 (5)	2 (4.3)	1

*BMI* body mass index, *TG* triglyceride, *ASA* American Society of Anesthesiologists classification, *ERCP* endoscopic retrograde cholangiopancreatography, *Others* drugs, pancreas divisum, autoimmune, intraductal papillary mucinous neoplasm, post-surgical procedure

Biliary pancreatitis was the more frequent etiology in the TG<200 mg/dL group in comparison with the TG>200/dL group (63 vs. 36.4%, *p*<0.001); this was opposite to alcoholic pancreatitis, which was the more frequent etiology in the TG≥200 mg/dL group compared with the TG<200 mg/dL group (40.3 vs. 13%, *p*<0.001).

### Laboratory and APACHE-II scores

Laboratory parameters on admission are described in Table 2. We found no differences between groups regarding the APACHE score and other relevant laboratory values.

**Table 2:** Laboratory and APACHE score of acute pancreatitis according to TG levels group

	TG<200 mg/dL	TG≥200 mg/dL	*P*
	*N*=200	*N*=47	
*Laboratory:*			
Amylase U/L, mean±SD	1194.86±1763.83	772.81±938.55	0.114
Triglycerides mg/dL, mean±SD	111.24±36.88	339.68±177.88	<0.001
Leukocytes 103/mL, mean±SD	13,914.29±6066.5	15,154.26±5322.23	0.086
Hematocrit %, mean±SD	42.74±5.44	41.79±6.69	0.596
Platelets 10^3^/mL, mean±SD	252.38±97.64	253.51±77.72	0.644
Creatinine mg/dL, mean±SD	1.03±0.49	1.15±0.58	0.159
BUN mg/dL	22.39±13.08	20.11±12.65	0.280
Bilirubin mg/dL, mean±SD	1.74±2.52	1.92±2.02	0.646
Alkaline phosphatase U/L, mean±SD	145.48±114.41	171.30±198.65	0.261
GGT mg/dL, mean±SD	261.83±363.68	588.02±685.33	0.004
AST U/L, mean±SD	202.78±274.74	186.02±327.16	0.717
ALT U/L, mean±SD	161.61±200.38	169.57±341.05	0.833
Albumin mg/dL, mean±SD	3.36±0.51	3.21±0.71	0.141
CRP mg/dL, mean±SD	5.43±7.74	8.78±11.11	0.069
APACHE II, mean±SD	7.02±3.82	6.85±3.51	0.791

*TG* triglyceride, *BUN* blood urea nitrogen, *GGT* gamma-glutamyl transpeptidase, *AST* aspartate aminotransferase, *ALT* alanine aminotransferase, *CRP* C-reactive protein, *APACHE II* Acute Physiology and Chronic Health Evaluation II score

### Local and systemic complications and severity

All parameters regarding local and systemic complications, CT severity indices, and severity of AP according to Atlanta definitions were worse in the TG≥200 mg/dL group than in the TG<200 mg/dL group, except in IPN (Table 3).

**Table 3:** Complications of acute pancreatitis according to TG levels group

	TG<200 mg/dL	TG≥200 mg/dL	*p*	OR (95% CI)
	*N*=200	*N*=47		
Respiratory failure, *N* (%)	20 (10)	10 (21,3)	0.033	2.43 (1.05–5.62)
Renal failure, *N* (%)	24 (12)	11 (23.4)	0.044	2.24 (1.01–4.98)
Cardiovascular failure, *N* (%)	15 (7.5)	9 (19.1)	0.025	2.92 (1.19–7.16)
Organ failure, *N* (%)	37 (18.5)	16 (34)	0.02	2.27 (1.13–4.58)
Persistent organ failure, *N* (%)	19 (9.5)	13 (27.7)	0.001	3.64 (1.65–8.07)
Multiple organ failure, *N* (%)	16 (8)	9 (19.1)	0.031	2.72 (1.12–6.62)
Persistent multiple organ failure, *N* (%)	14 (7)	8 (17)	0.044	2.73 (1.07–6.94)
SIRS, *N* (%)	70 (35%)	25 (53,2)	0.021	2.11 (1.11–4.01)
Persistent SIRS, N (%)	44 (22.2)	17 (37.8)	0.043	2.01 (1.01–3.98)
*ATLANTA classification, N (%)*			<0.001	
MAP	119 (59.5)	15 (31.9)		
MSAP	63 (31.5)	19 (40.4)		
SAP	18 (9)	13 (27.7)		
ATLANTA MSAP and SAP, *N* (%)	81 (40.5)	32 (68.1)	0.001	3.13 (1.59–6.16)
Pancreatic necrosis, *N* (%)	68 (34)	30 (63.8)	<0.001	3.43 (1.77–6.65)
Pancreatic necrosis infection, *N* (%)	16 (23.5)	8 (26.7)	0.739	1.18 (0.44–3.16)
Invasive procedure against necrosis, *N* (%)	19 (9.5)	12 (25.5)	0.003	3.27 (1.46–7.33)
CT severity index, mean±SD	3.24±2.49	4.87±2.84	0.001	
Modified CT severity index, mean±SD	4.94±2.82	6.15±3.09	0.022	
Admission to ICU, *N* (%)	19 (9.5)	13 (27.7)	0.001	3.64 (1.65–8.07)
Hospital length of stay (days), mean±SD	17.81±22.97	27.04±29.91	0.020	
Mortality, *N* (%)	7 (3.5)	5 (10.6)	0.056	3.28 (0.99–10.85)

*TG* triglyceride, *SIRS* systemic inflammatory response syndrome, *ATLANTA* Atlanta Classification of the severity, *MAP* mild acute pancreatitis, *MSAP* moderately severe acute pancreatitis, *SAP* severe acute pancreatitis, *CT* computed tomography, *ICU* intensive care unit

*Invasive procedure against necrosis include interventional radiological procedure, endoscopic procedure, and surgical intervention

We carried out an independent analysis according to sex and found no differences in the association of TG levels>200 mg/dL with the outcomes of AP observed in our total population.

### Hospital stay and mortality

The need for admission to the ICU was higher in the TG≥200 mg/dL group than in the group with TG<200 mg/dL (27.7 vs. 9.5%, *p*=0.001, OR: 3.64).

The length of hospital stay was higher in the TG≥200 mg/dL group (27.04±29.91 vs. 17.81±22.97, *p*=0.02).

Mortality was higher in the TG≥200 mg/dL group (10.6%) than in the TG<200 mg/dL group (3.5%), although these differences were not statistically significant.

### Multivariable analysis for outcomes of AP

In the multivariable logistic regression analysis to evaluate the association between TG levels≥200 mg/dL and other risk factors for each outcome of AP, we found that TG levels≥200 mg/dL were independently associated with respiratory failure (OR: 3.37), renal failure (OR: 3.56), cardiovascular failure (OR: 3.03), organ failure (OR: 3.56), persistent organ failure (OR: 4.65), multiple organ failure (OR: 2.83), pancreatic necrosis (OR: 2.92), severe pancreatitis (OR: 4.25), and admission to the ICU (OR: 3.57), as shown in Table 4. We did not find an association between TG levels≥200 mg/dL and IPN or mortality.

**Table 4:** Univariate and multivariable analysis to evaluate the association between TG level≥200 mg/dL and other risk factors for each outcome of acute pancreatitis

	Univariate analysis		Multivariable analysis	
	OR (95% CI)	*p* value	OR (95% CI)	*p* value
*Respiratory failure*				
Age (≥ 65 years)	2.46 (1.01–5.99)	0.046	2.51 (0.9–7.01)	0.078
Higher blood pressure	2.76 (1.14–6.69)	0.025	2.03 (0.77–5.32)	0.151
TGs (≥ 200 mg/dL)	2.43 (1.05–5.62)	0.038	3.37 (1.35–8.43)	0.009
*Renal failure*				
Diabetes	2.95 (1.39–6.23)	0.005	1.87 (0.82–4.25)	0.135
Higher blood pressure	2.9 (1.26–6.68)	0.012	1.85 (0.73–4.65)	0.194
Cardiovascular disease	2.34 (1.11–4.95)	0.026	1.53 (0.64–3.73)	0.34
Chronic kidney disease	4.89 (1.93–12.43)	0.001	2.79 (0.93–8.37)	0.067
Biliary etiology	2.79 (1.21–6.43)	0.016	2.43 (0.95–6.25)	0.065
TGs (≥ 200 mg/dL)	2.24 (1.01–4.98)	0.048	3.56 (1.43–8.86)	0.006
*Cardiovascular failure*				
Diabetes	3.17 (1.33–7.52)	0.009	2.52 (1.02–6.19)	0.044
Higher blood pressure	3.15 (1.14–8.72)	0.028	2.74 (0.95–7.89)	0.062
TGs (≥ 200 mg/dL)	2.92 (1.19–7.16)	0.019	3.03 (1.19–7.66)	0.02
*Organ failure*				
Diabetes	2.49 (1.29–4.81)	0.007	1.62 (0.78–3.33)	0.193
Higher blood pressure	3.21 (1.59–6.48)	0.001	2.28 (1.06–4.89)	0.035
Cardiovascular disease	2.02 (1.05–3.9)	0.036	1.49 (0.7–3.2)	0.297
Chronic kidney disease	3.24 (1.33–7.88)	0.01	1.71 (0.61–4.77)	0.304
Biliary etiology	2.72 (1.37–5.4)	0.004	2.65 (1.21–5.8)	0.015
TGs (≥ 200 mg/dL)	2.27 (1.13–4.58)	0.022	3.56 (1.59–7.98)	0.002
*Persistent organ failure*				
Diabetes	3.02 (1.39–6.55)	0.005	2.11 (0.91–4.88)	0.08
Higher blood pressure	3.05 (1.26–7.35)	0.013	2.61 (1.01–6.75)	0.048
Chronic kidney disease	3.48 (1.31–9.29)	0.013	3.24 (1.12–9.38)	0.03
TGs (≥ 200 mg/dL)	3.64 (1.65–8.07)	0.001	4.65 (1.96–11.03)	<0.001
*Multiple organ failure*				
Diabetes	3.53 (1.51–8.26)	0.004	2.8 (1.16–6.76)	0.022
Higher blood pressure	3.34 (1.21–9.21)	0.02	2.83 (0.99–8.13)	0.053
TGs (≥ 200 mg/dL)	2.72 (1.12–6.62)	0.027	2.83 (1.12–7.13)	0.028
*Pancreatic necrosis*				
Sex Male	4.21 (2.38–7.44)	<0.001	3.85 (2.11–7.01)	<0.001
Alcohol etiology	2.21 (1.15–4.25)	0.018	1.05 (0.5–2.19)	0.894
TGs (≥ 200 mg/dL)	3.43 (1.77–6.65)	<0.001	2.92 (1.43–5.96)	0.003
*ATLANTA Severe AP*				
Diabetes	2.74 (1.25–6.02)	0.012	2.21 (0.96–5.09)	0.062
Higher blood pressure	2.39 (1.02–5.58)	0.044	2.09 (0.85–5.19)	0.109
Cardiovascular disease	3.65 (1.36–9.75)	0.01	1.47 (0.62–3.52)	0.382
TGs (≥ 200 mg/dL)	3.13 (1.59–6.16)	0.001	4.25 (1.83–9.87)	0.001
*Admission to ICU*				
Diabetes	2.2 (1.01–4.84)	0.049	2.14 (0.95–4.79)	0.065
TGs (≥ 200 mg/dL)	3.64 (1.65–8.07)	0.001	3.57 (1.59–7.97)	0.002

*ATLANTA* Atlanta Classification of the severity, *AP* acute pancreatitis, *ICU* intensive care unit, *TG* triglycerides, *OR*: odds ratio, *CI* confidence interval

## Discussion

Our study observed that patients with TG levels≥200 mg/dL measured in the first 24 h after admission for AP had a worse clinical outcome. Serum TG levels≥200 mg/dL were associated with respiratory failure, cardiovascular failure, organ failure, persistent organ failure, multiple organ failure, pancreatic necrosis, pancreatitis severity, and admission to the ICU.

The role of elevated TG levels in patients with non-HTG syndrome-induced AP is unknown. Some authors have reported that elevated TG levels are related to severe forms of acute necrotizing pancreatitis, concluding that elevated TG levels are an independent factor for severe AP [15, 29–31]. However, other studies show no significant relationship between elevated TG levels and the severity of AP, and one of the hypotheses is that elevated TG levels represent only a secondary manifestation of AP [32–34].

The mechanism explaining why elevated TG levels are related to severe AP is still unknown. However, several pathogenic theories have been proposed: in the early stages of AP, the release of serum catecholamines and glucagon, as well as pancreatic lipases, in response to stress accelerates the decomposition of the adipose tissue with the consequent release of triglycerides as its degradation products (free fatty acids, FFA) through the systemic circulation [35]. It is well-known that excess FFA in the circulation induces direct tissue damage [36–38], positive regulation of cytokines, and activation of inflammatory cascades that predispose to organ failure [39]. Furthermore, under conditions of excess FFA, the plasma’s high viscosity could also cause capillary ischemia of the pancreatic parenchyma [40]. Other explanations could be related to the release of cytokines secondary to AP, especially in necrotizing AP. Interleukin-6 is one of the most frequent cytokines in AP. Higher concentrations of IL-6 are related to severe forms of AP, worsening the clinical course of the disease [41]. Additionally, IL-6 plays a role in the release of triglycerides from hepatocytes through the circulation [42]. The release of higher cytokine concentrations in severe AP could explain the early elevated TG levels and the consequently higher incidence of complications in this group of patients.

Additionally, the extravasation of FFA through the alveolar-capillary membrane may explain the lung injury in AP patients with elevated TG levels [43, 44]. Our study showed that respiratory failure and cardiovascular failure were higher in patients with a high TG level at admission.

Elevated TG levels have also been described as an independent risk factor for the development of acute renal failure in patients with AP [45], probably explained by the fact that the triglycerides deposited around the renal tubules will react with pancreatic lipase, which can directly damage the renal parenchyma and produce a high level of free fatty acids around the renal cells [46]. In our study, patients in the TG≥200 mg/dL group had a higher incidence of acute renal failure.

Elevated TG levels have been described as an independent predictor of pancreatic necrosis [47], and they are also related to the extension of parenchymal necrosis [35]. We found similar results in our cohort regarding the relationship with pancreatic parenchymal necrosis. However, the findings were not the same for the occurrence of infectious pancreatic necrosis (IPN) since its mechanism involves other crucial risk factors, such as bacterial translocation from the intestinal tract, use of total parenteral nutrition, or extrapancreatic source of infections [48].

In our cohort, the TG≥200 mg/dL group did not experience significant mortality compared with that in the TG<200 mg/dL group. One explanation relies on the low mortality rate in our entire cohort, making it difficult to find differences between groups.

Identifying the mechanisms by which TG levels increase in acute pancreatitis and how they cause tissue damage will help develop new treatment strategies for severe forms of AP. Studies carried out in AP induced by HTG suggest that early removal of triglycerides and toxic free fatty acids may be advantageous; however, high-quality evidence is still missing in the literature [49–52].

There are some limitations to our study. First, TG values were measured within 24 h after admission to the hospital; thus, the time between the onset of symptoms and serum TG measurement is not the same in all patients. However, to diminish bias, we selected patients with no more than 72 h from the onset of symptoms until admission. Second, the TG values of the patients before the AP episode were not available, which could have influenced the values obtained on admission, making it difficult to discern whether the TG levels were a consequence of the AP or a secondary, previous conditioning disease. Third, the fact that the basal characteristics of both groups differed in sex, alcohol etiology, and BMI could be a source of bias regarding the higher incidence of complications in this type of patient. For this reason, we performed a multivariable analysis to determine whether TG levels≥200 mg/dL were associated with a worse prognosis compared to other risk factors.

In summary, our prospective study found that TG levels≥200 mg/dL at admission in patients with AP are associated with greater local and systemic complications. Moreover, elevated serum triglycerides are an independent risk factor for complications and severe AP. Therefore, measuring TG levels early in patients with AP could help identify these at-risk patients.

## Supplementary Information

Below is the link to the electronic supplementary material.

Supplementary file1 (DOCX 77 KB)
