# Supplementary material for: Elevated Serum Triglyceride Levels in Acute Pancreatitis: A Parameter to be Measured and Considered Early
Source: World J Surg. 2022 Mar 30;46(7):1758–67. doi: 10.1007/s00268-022-06533-w (PMC9174303; doi:10.1007/s00268-022-06533-w)
Supplement: Supplementary file 1 — Supplementary file1 (DOCX 77 KB) [file 268_2022_6533_MOESM1_ESM.docx]

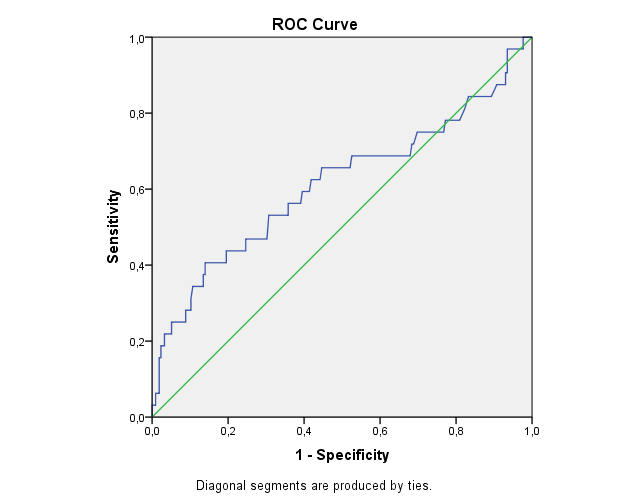


Fig 1. ROC curve of triglycerides in predicting the Organ failure in patients with acute pancreatitis. AUC: 0.613, p: 0.04, Youden's Index: 205.5 mg/dL.


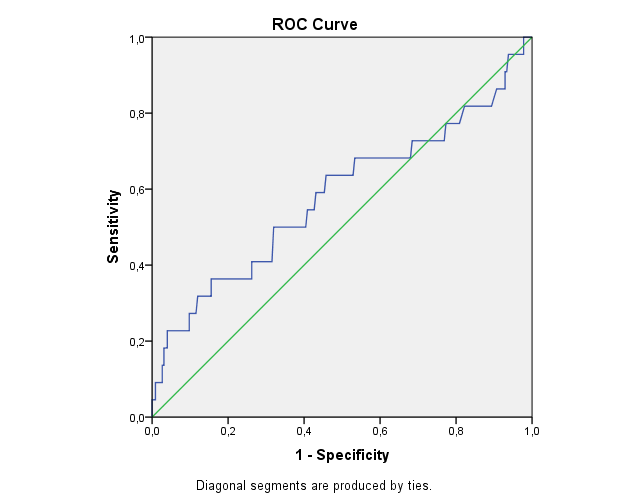


Fig 2. ROC curve of triglycerides in predicting the multiple organ failure in patients with acute pancreatitis. AUC: 0.591, p: 0.041, Youden's Index: 205.5 mg/dL.


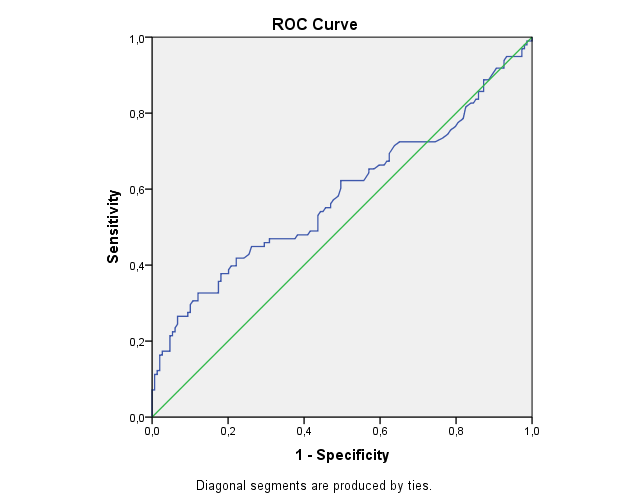


Fig 3. ROC curve of triglycerides in predicting the pancreatic necrosis. AUC: 0.583, p: 0.027, Youden's Index: 202,5 mg/dL.


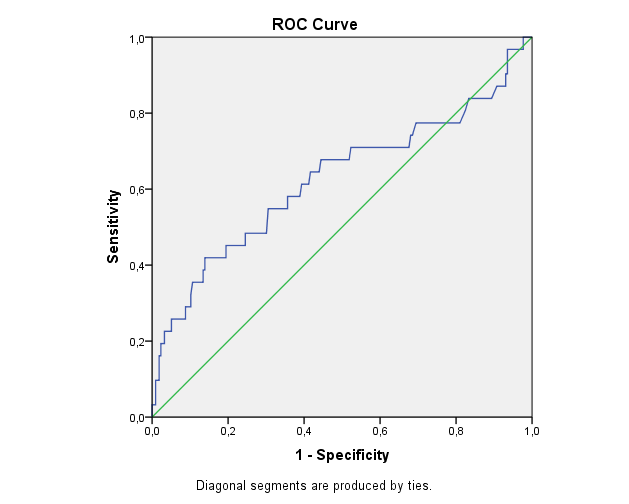


Fig 4. ROC curve of triglycerides in predicting severe acute pancreatitis. AUC: 0.626, p: 0.023, Youden's Index: 204 mg/dL.


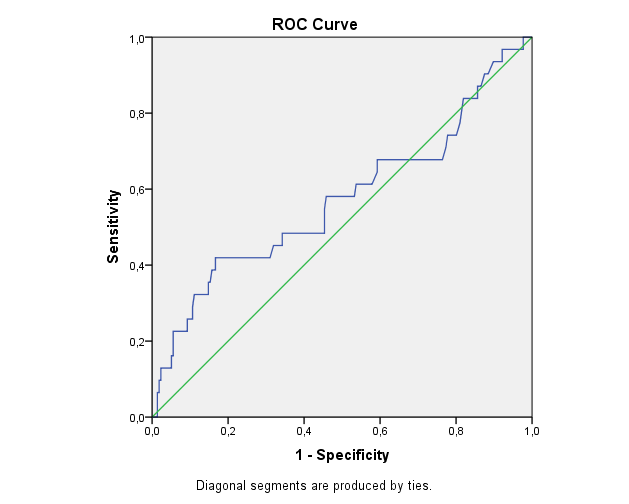


Fig 5. ROC curve of triglycerides in predicting admission to ICU. AUC: 0.601, p: 0.034, Youden's Index: 205.5 mg/dL.
